# Evaluación de la resistencia adhesiva de una resina compuesta a dentina tratada con clorhexidina y terapia fotodinámica. estudio *in vitro*

**DOI:** 10.21142/2523-2754-1101-2023-142

**Published:** 2023-03-26

**Authors:** Joaquín Enrique Huarote Fernández, Jocelyn Graciela Lugo Varillas

**Affiliations:** 1 Carrera de Estomatología, Universidad Científica del Sur. Lima, Perú. joaquinhuarote@hotmail.com Universidad Científica del Sur Carrera de Estomatología Universidad Científica del Sur Lima Peru joaquinhuarote@hotmail.com; 2 Departamento de Ciencias de la Salud, Carrera de Estomatología, Universidad Científica del Sur. Lima, Perú. jlugo@cientifica.edu.pe Universidad Científica del Sur Departamento de Ciencias de la Salud Carrera de Estomatología Universidad Científica del Sur Lima Peru jlugo@cientifica.edu.pe

**Keywords:** resistencia adhesiva, clorhexidina, terapia fotodinámica, resina compuesta, strenght bond, clorhexidine, photodinamic therapy, composite

## Abstract

**Introducción:**

La terapia fotodinámica consiste en la aplicación de una luz con una adecuada longitud de onda sobre las cavidades, previa a la colocación de un agente fotosensibilizador, el cual tiene como finalidad eliminar restos de microorganismos remanentes luego de la instrumentación.

**Objetivo:**

Evaluar la resistencia adhesiva a dentina tratada con clorhexidina al 2% y terapia fotodinámica con diodo 660 nm, previa a la inserción de resina compuesta.

**Materiales y métodos:**

En este estudio experimental in vitro, se recolectó 60 incisivos mandibulares bovinos que recibieron un desgaste de la dentina. Las muestras se dividieron en 3 grupos (20 c/u): control (sin terapia), clorhexidina al 2% y láser diodo 660nm (fotosensibilizador de azul de metileno). La resistencia adhesiva fue medida por una prueba de cizallamiento comparado con la Prueba de Kruskal-Wallis y post-hoc de comparación por parejas.

**Resultados:**

Los valores promedio de mayor a menor resultaron con la CHX (14,82 ± 3,14), seguido del láser diodo 660 nm (14,77 ± 4,02) y el grupo control (9,25 ± 1,16). Los grupos láser diodo 660 nm y CHX al 2% obtuvieron valores similares (P > 0,05), pero significativamente más altos con respecto al grupo control (P < 0,001). **Conclusión:** La terapia fotodinámica aumentó la resistencia adhesiva, al igual que la clorhexidina, en comparación con el grupo control.

## Abstract (en)

**Introduction:**

Photodynamic therapy consists of the application of a light with an adequate wavelength on the cavities prior to the application of a photosensitizing agent, with the purpose of eliminating remnants of microorganisms remaining after instrumentation.

**Objective:**

Evaluate the adhesive resistance to dentin treated with 2% chlorhexidine and photodynamic therapy with a 660nm diode prior to the insertion of composite resin.

**Materials an Methods:**

In this in vitro experimental study, 60 bovine mandibular incisors were collected, After dentin wear, the samples were divided into 3 groups, with 20 samples per group: control group (no therapy was applied), 2% chlorhexidine, and 660nm diode laser (photosensitizer: methylene blue). Adhesive strength test was measured by shear test compared to kruskall-wallis test and post-hoc pairwise comparison.

**Results:**

The average values from highest to lowest result with the CHX (14.82 ± 3.14), followed by the 660nm diode laser (14.77 ± 4.02) and the control group (9.25 ± 1.16). Similar groups of 660 nm diode laser and 2% CHX changed values (P>0.05), but significantly higher than the control group (P<0.001). **Conclusion:** Photodynamic therapy increased adhesive resistance as well as chlorhexidine, both therapies presented an increase in adhesive resistance compared to the control group.

## INTRODUCCIÓN

El uso de restauraciones de resina compuesta (RC) aumentó de manera significativa debido a sus características estéticas y óptimas en las preparaciones dentales no invasivas ^1^. El éxito del tratamiento depende mucho de la interfaz adhesiva, factor preocupante a largo plazo ^2^. Clínicamente la falla de unión disminuye la durabilidad de la restauración adhesiva, asociada frecuentemente con la decoloración marginal, la desadaptación, los defectos marginales y la pérdida de retención de restauraciones adhesivas ^2^.

La adhesión es la unión del diente y el material restaurador. Esta unión debería ser lo suficientemente resistente y duradera en esta interfase ^3^, pero posee limitaciones como la unión a dentina, que resulta más crítica que la del esmalte debido a la presencia de túbulos dentinarios, por lo que es un medio más permeable y acuoso ^4^.

Las diferentes técnicas antimicrobianas utilizadas en las cavidades dentales son el hipoclorito de sodio (NaOCl) al 2,5% y la clorhexidina (CHX) al 2%. Ambos son agentes antibacterianos usados de manera regular contra diferentes especies, aunque se reportan algunas desventajas y resultados contradictorios después de su colocación ^5^.

La terapia fotodinámica (TFD) es otra técnica antimicrobiana novedosa utilizada con éxito contra la caries ^6^. Su mecanismo incluye agentes fotosensibilizadores colorantes como el azul de metileno y el verde de indocianina ^7^. Un ejemplo es el láser diodo 660 nm, cuya longitud de onda ha demostrado ser bactericida ^8^. También tiene un menor costo en comparación con otros láseres y es una fuente de luz usada en una gran variedad de estudios ^9^.

Los láseres de diodo, también denominados semiconductores, son una combinación de aluminio, galio y arsénico que se usan para transformar la energía eléctrica en lumínica. Hay dos tipos, según su potencia según su longitud de onda: baja (635 nm y 830 nm) y alta (810 nm y 980 nm). En cuanto a su aplicación clínica, los de baja potencia son principalmente bioestimuladores, antiinflamatorios y bactericidas ^10^. Estos se aplican en el área de interés y se irradian con una luz visible, lo cual produce radicales libres de oxígeno y elimina los microorganismos cariogénicos comunes en cavidad oral, como los *Streptococcus mutans* y *Lactobacillus acidophilus*^6^.

Los resultados sobre resistencia adhesiva en restauraciones de resina compuesta realizadas sin la utilización de CHX al 2% previa al sistema adhesivo obtuvieron mayores valores en comparación con las que la utilizaron ^11^. También se han encontrado valores de resistencia de unión significativamente más altos para los sistemas adhesivos que recibieron irradiación de láser Nd:YAG antes de la polimerización ^12^. Un estudio halló que los valores medios de la fuerza de unión fueron más altos en los grupos tratados con láser diodo que sus homólogos sin láser ^13^.

Ante los anteriores resultados, se considera una alternativa de desinfección diferente a las convencionales -que podrían lograr mejores valores de resistencia adhesiva-, el realizar la descontaminación de cavidades dentales antes del protocolo de adhesión con la TFD ^(14, 15)^. Por ello, el propósito del estudio *in vitro* será comparar la resistencia adhesiva a dentina tratada con clorhexidina y terapia fotodinámica con diodo 660 nm previa a la inserción de resina compuesta. La hipótesis nula fue que existió diferencias en la resistencia adhesiva de una resina compuesta a dentina entre ambos tratamientos.

## MATERIALES Y MÉTODOS

El presente estudio fue aceptado por el comité de etica de la Universidad Cientifica el Sur y por la carrera de estomatologia con el número de aprobación 384-2020-PRE99.

Este estudio experimental *in vitro* estuvo conformado por una muestra de 60 incisivos mandibulares bovinos, los cuales se obtuvieron de un centro de acopio ganadero de Lima (Camal de Yerbateros®). Luego, fueron distribuidas en 3 grupos (20 muestras c/u), un grupo control y dos grupos experimentales (CHX y TFD).

La muestra incluidas fueron incisivos mandibulares bovinos sin grietas, sin erosiones y sin lesiones cariosas, extraídos de un animal donador bovino no mayor de 5 años en un plazo menor a siete días, y que cumplieron con los requisitos de presentar superficies vestibulares planas. En cuanto a los materiales, como el adhesivo, el agente fotosensibilizador y el ácido fosfórico, estuvieron sellados y vigentes. Se excluyeron dientes incisivos con presencia de líneas de fractura, con alguna pigmentación blanca y con defectos de discromía.

### Obtención de número muestral

Un estudio piloto fue realizado con cinco muestras por cada grupo de estudio (10% de la muestra de un estudio previo) ^4^. Para obtener el tamaño muestral, se utilizó una fórmula de comparación de dos promedios, con un poder estadístico del 80%, un nivel de confianza del 95% y una precisión de 6,27. El tamaño por grupo fue pequeño, por lo que los investigadores decidieron aumentarlo a 20 muestras por cada grupo.

### Obtención de los discos de dentina

Un total de 60 incisivos mandibulares bovinos fueron almacenados en timol al 0,1% (Farmacia Universal®) en un frasco de vidrio de 500 ml, donde fueron completamente sumergidos y refrigerados (4 °C) por un periodo de 1 mes, en promedio. Los dientes fueron limpiados con curetas periodontales Gracey estándar (Everedge 2.0 Hu-Friedy®) y pulidos con piedra pómez (Comindent®) usando una escobilla tipo Robinson (Woodpecker®) en baja rotación. El lavado se realizó con agua destilada.

Los dientes se cortaron en el límite amelocemental con la ayuda de un disco diamantado de baja velocidad (MDC Dental®). La pulpa dentaria fue retirada con una lima 80 (Dentsply Maillefer K-file®) y se selló la entrada del conducto con resina epóxica. Luego, se almacenaron las muestras en agua destilada y fueron refrigeradas a 4 °C por 7 días. 

Las zonas vestibulares de las coronas fueron fijadas en láminas de cera blanca para ortodoncia (Vitis Orthodontic Dentaid®) e insertadas de manera individual en tubos de PVC (Pavco Vinduit®) de 15 mm de altura y 25 mm de diámetro. La resina epóxica fue aplicada en el interior del tubo de PVC para su fijación con la cara vestibular expuesta. Dicha resina presentó baja liberación de calor durante su polimerización.

Al terminar la polimerización, la superficie vestibular se pulió consecutivamente con una máquina pulidora (Ecomet® 3 - Buehler Ltd., IL, EUA) a 300 rpm, con papel de lija abrasiva de carburo de sílice n.o 120, n.o 240, n.o 400 y n.o 600 (Norton®), hasta visualizar la dentina. En cada cambio de papel, los especímenes se lavaron con una máquina de ultrasonido en agua destilada por 1 minuto. Finalmente, la muestra se pulió con un disco de felpa (Macrodent®).

**Figura 1:**
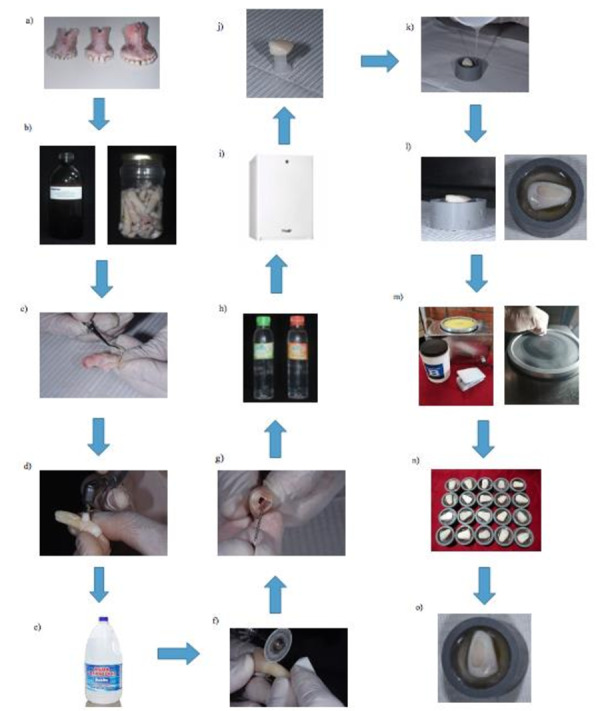
Diagrama de flujo de preparación de las muestras. **a)** Dientes incisivos bovinos. **b)** Dientes bovinos sumergidos en un frasco de timol al 0,1%, refrigerados por un promedio de 1 mes. **c)** Limpieza de los dientes con curetas periodontales. **d)** Dientes siendo pulidos con piedra pómez y agua destilada en baja rotación con la ayuda de una escobilla tipo Robinson. **e)** Los dientes se lavaron con agua destilada. **f)** Raíces de los dientes cortadas con un disco diamantado en baja velocidad (mcd dental). **g)** Retiro de la pulpa con una lima de 80 mm. **h)** Se selló el conducto con resina epóxica. **i)** Las muestras se almacenaron en agua destilada refrigeradas a 4 °C por siete días. **j)** Después de siete días, las muestras dejaron de ser refrigeradas y se fijaron con cera blanca en el centro de un tubo de PVC. **k)** Se aplicó resina epóxica para la fijación de dichas muestras. **l)** Muestras insertadas en el tubo de PVC y fijadas con resina epóxica. **m)** Se desgastó la cara vestibular con una máquina de desgaste hasta visualizar la dentina. **n)** Muestras con la superficie de dentina expuesta. **o)** Muestra preparada.

En la Fig. 1 se puede observar de manera detallada la preparación de los discos de dentina.

### Protocolo de irradiación

**Figura 2:**
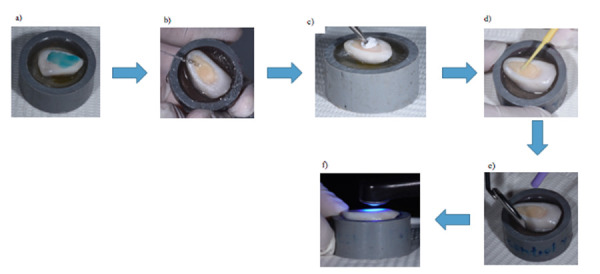
Diagrama de flujo de preparación del grupo control**. a)** Aplicación en dentina de ácido fosfórico al 37% por 15’’; **b)** Lavado con agua por 30’’, **c)** Secado de la superficie de unión con papel filtro; **d)** Colocación del adhesivo frotando por 20’’; **e)** Aplicación de aire indirecto para eliminar el solvente por 20’’; **f)** Fotopolimerización del adhesivo.

La muestra total se dividió en tres grupos de 20 especímenes cada uno. En el grupo 1 (control), los especímenes fueron tratados con ácido fosfórico al 37% (Condac FGM®) en dentina (15’’) y, enseguida, fueron lavados con agua destilada (30’’). Se procedió a secar con bolitas de papel filtro (Filtrantes del Perú®) y se aplicó adhesivo *etch and rinse* de 2 pasos, Single Bond (Adper® Single Bond 2, 3M ESPE, 2510 Conway Avenue St. Paul). La aplicación se realizó con un microbrush (TPC®) y se frotó sobre la dentina (20”); luego, se fotopolimerizó con una unidad led de Valo (VALO® cordless 505 West Ultradent Drive South Jordan, UT 84095) (20”), según el fabricante. Al final, se colocaron bloques de resina (Filtek® Z350XT, 3M ESPE,2510 Conway Avenue St. Paul) y se fotopolimerizó (20”) cada uno (Fig. 2).

**Figura 3:**
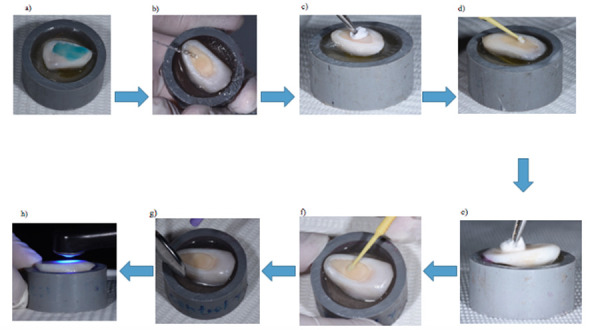
Diagrama de flujo de protocolo de preparación para muestras del grupo clorhexidina. **a)** Aplicación de ácido fosfórico al 37% por 15” en dentina, después se lavó por un promedio de 30”; **b)** Se retiró el ácido fosfórico al 37% con agua por 30”; **c)** Se secó la superficie de unión con papel filtro; **d)** Se aplicó CHX al 2% por 1’ con microbrush (frotado en toda la superficie de dentina); **e)** Se secó ligeramente la CHX con una bolita de algodón; **f)** Se aplicó el adhesivo, frotando por 20”; **g)** aplicación de aire indirecto por 20” para eliminar el solvente; **h)** Se fotopolimerizó el adhesivo.

En el grupo 2, después de la aplicación del ácido fosfórico al 37% (Condac FGM®) (15”) en dentina, se lavó (30”) las 20 muestras y fueron frotadas con clorhexidina al 2% (Maquira®) usando un microbrush (TPC®) (1’). Luego, se procedió a secar con papel de filtro (Filtrantes del Perú®). Se aplicó un adhesivo *etch and rinse* de 2 pasos single bond (Adper® Single Bond 2, 3M ESPE,2510 Conway Avenue St. Paul) con un microbrush (TPC®), este se frotó sobre la dentina (20”), después se volatizó el solvente (20”) y se fotopolimerizó con una unidad led de VALO (VALO® cordless 505 West Ultradent Drive South Jordan, UT 84095) (20”) según el fabricante. Finalmente, se colocaron bloques de resina (Filtek® Z350XT,3M ESPE,2510 Conway Avenue St Paul) y se polimerizó (20”) cada bloque (Fig. 3).

**Figura 4:**
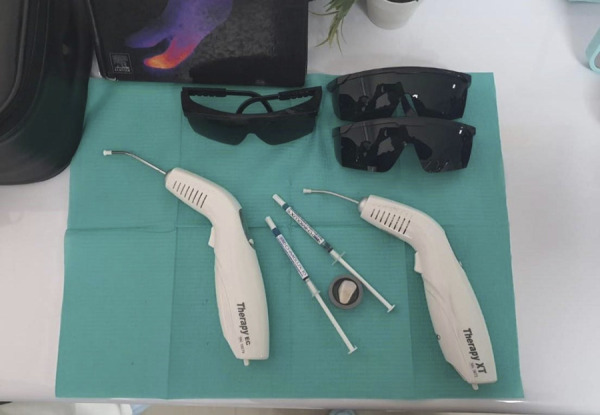
Fotografías del estudio. Láser diodo 660nm therapy XT

**Figura 5:**
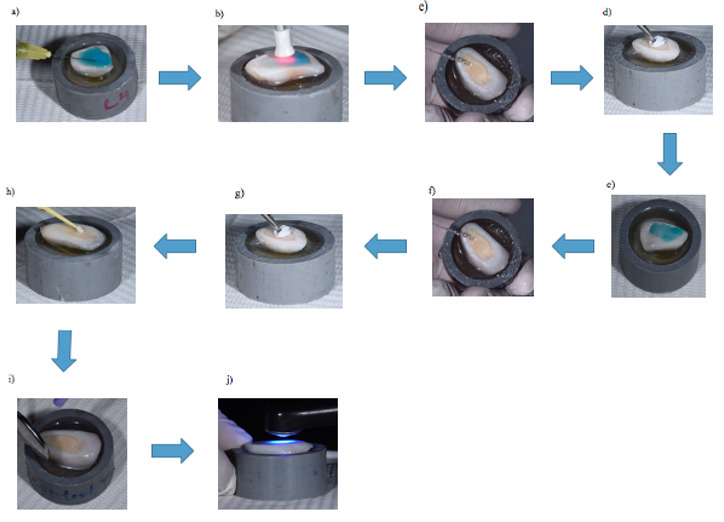
Diagrama de flujo de protocolo de preparación para muestras del grupo TFD diodo 660nm. **a)** Se aplicó el fotosensibilizador azul de metileno al 0.001% por 5’ (tiempo de incubación); **b)** Después de dicho periodo se aplicó una fuente de luz sobre el azul de metileno con una longitud de onda de 660nm , área de trabajo de 0,028cm2, potencia 100 mW, densidad de energía de 320 J/cm2, exposición de 90”, energía total de 9 J; **c)** Retiro del fotosensibilizador con un chorro de agua por un promedio de 30”; **d)** Secado de la superficie con papel filtro; **e)** Aplicación de ácido fosfórico al 37% por 15” en dentina; **f)** Retiro del ácido fosfórico con un chorro de agua por 30”; **g)** Secado de la superficie con papel filtro; **h)** Aplicación de adhesivo y frotado por 20”; **i)** Aplicación de aire indirecto por 20” para eliminar el solvente; **j)** fotopolimerización del adhesivo.

En el grupo 3, 20 muestras fueron tratadas con terapia fotodinámica. Primero, se aplicó el fotosensibilizador azul de metileno al 0,001% (Chimiolux® 5, DMC Equipamentos, San Carlos, SP, Brasil) (5’) (tiempo de incubación) (Fig. 4); después de ese periodo, se aplicó una fuente de luz sobre el azul de metileno con una longitud de onda de 660 nm, con un área de trabajo de 0,028 cm^2^, potencia de 100 mW, densidad de energía de 320 J/cm^2^, exposición de 90” y energía total de 9 J. Terminada la irradiación, se retiró el fotosensibilizador con un chorro de agua (30”) y se realizó el protocolo adhesivo antes mencionado (Fig. 5).

En la Tabla 1 se puede ver el lote y la composición de cada material utilizado.

**Tabla 1:** Composición y lote de los materiales empleados en el estudio

Marca	Código	Fabricante	Tamaño/forma	Lote	Composición		
Resina nanohibrida Filtek Z350 XT	Z350	3M, ESPE, St. Paul, Mn, EE. UU.	A2E	7018A2B	Monómero	Relleno		
					Bis-GMA, UDMA, Bis-EMA, TEGDMA, PEGDMA	Composición	Mass % (Vol %)
						SiO2(20nm), ZrO2 (4-11nm), agregado ZrO2/SiO2, aglomerado (0.6-10um)	73(56)
Adhesivo (Adper Single Bond 2)	Single Bond 2	3M, ESPE, St. Paul, Mn, EE. UU.	----------	51202	BisGMA, HEMA	Dimetacrilatos, etanol, agua, copolímero funcional de metacrilato de ácido poliacrílico y ácido politacónico	15%
Clorhexidina 2% (maquira)	----------	Maquira	----------	577117	----------	gluconato de clorhexidina	100 mL
Fotosensibilizador azul de metileno (Chemiolux)	----------	DMC	----------	----------	----------	agua purificada y azul de metileno	1,5 mL

### Ensayo de cizallamiento

**Figura 6:**
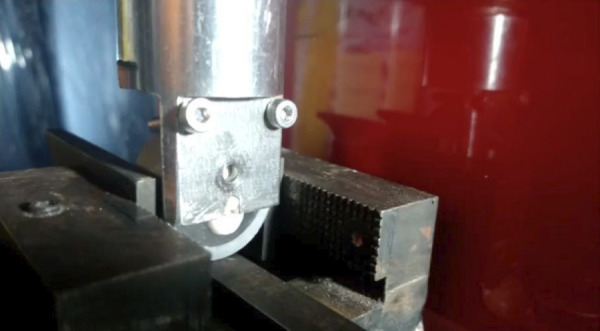
Fotografías del estudio. Máquina de cizallamiento

La prueba de cizallamiento se realizó luego de 24 horas de almacenamiento, durante las cuales las muestras fueron adaptadas al dispositivo para la ejecución del test de cizallamiento en una máquina digital de ensayos universales CMT-5 L (Fig. 6). Un borde entallado se colocó de manera paralela al eje longitudinal del diente apoyado sobre el cilindro de resina, el cual se realizó con una velocidad de 1 mm/min y fuerza de 50 N; luego se convirtió a MPa.

### Análisis estadístico

La estadística descriptiva incluyó media, desviación estándar, varianza, mínimo, máximo, mediana y rango. La normalidad fue evaluada con la prueba de Shapiro-Wilk. Las comparaciones entre grupos fueron analizadas con la prueba de Kruskall-Wallis, con *post-hoc* de comparación por parejas, y la prueba de U de Mann Whitney para comparar 2 grupos con datos independientes. Para el análisis de los datos, se usó el programa IBM SPSS® v.21 (SPSS Inc.Chicago, IL, EE. UU.) a un nivel de significancia de 0,05.

## RESULTADOS

**Figura 7:**
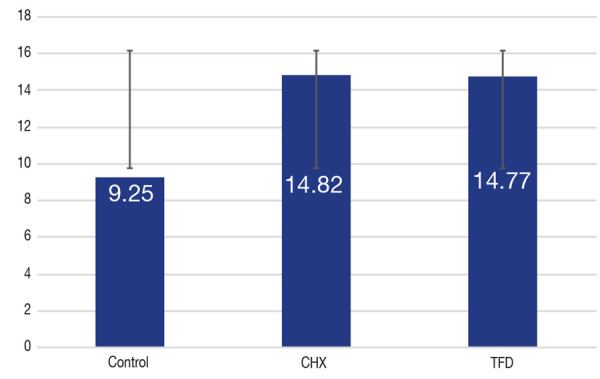
Comparación de la resistencia adhesiva de los tres grupos experimentales (desviación estándar y barras de error).

En la Tabla 2 y la Figura 7 se muestran los resultados de resistencia de unión de los grupos de estudio en los cuales los valores promedios, de mayor a menor, resultaron con la CHX (14,82 ± 3,14), seguido de la TFD (14,77 ± 4,02) y el grupo control (9,25 ± 1,16).

**Tabla 2:** Evaluación descriptiva de la resistencia adhesiva de una resina compuesta a dentina tratada con CHX al 2% y TFD

Grupos de estudio	Promedio	Desviación estándar	Mediana	Mínimo	Máximo
Control	9,25	1,16	9,18	7,02	11,9
CHX	14,82	3,14	14,45	10,56	22,47
Láser	14,77	4,023	15,28	8,12	22,98

En la Tabla 3 se observa la comparación de resistencia adhesiva entre los grupos. Los grupos TFD (15,28 [22,98; 9,73]) y CHX al 2% (14,45 [17,28; 11,11]) obtuvieron valores similares (P > 0,05), pero significativamente más altos que respecto del grupo control (9,18 [9,47; 9,09]) (P < 0,001).

**Tabla 3:** Comparación de la resistencia adhesiva de una resina compuesta a dentina tratada CHX al 2% y TFD

Grupos de estudio	Resistencia adhesiva	P valor
	Mediana [Q1, Q3]	
Control	9,18 [9,47; 9,09]b	< 0,001
CHX al 2%	14,45 [17,28; 11,11] a	
Láser diodo 660 nm	15,28 [22,98; 9,73]a

Letras distintas indican diferencias significativas. Prueba de Kruskal Wallis con post-hoc de comparación por parejas.

## DISCUSIÓN

El éxito de las restauraciones adhesivas directas depende de muchos factores como la reducción de microrganismos con procedimientos coadyuvantes que eviten infecciones dentarias luego de la remoción de dentina infectada. Uno de los coadyuvantes principales es la CHX; sin embargo, existe también la TFD, que consiste en el uso de agentes fotosensibilizantes que interactúan con luz láser, aunque dañan la membrana celular y el ADN del microorganismo, lo que provoca su muerte ^4^.

Por otro lado, existen diferentes pruebas de resistencia adhesiva en la interfase restauración-diente que permite inferir una longevidad de las restauraciones, como la prueba a la tracción, microtracción, cizallamiento y microcizallamiento. En el presente estudio, se escogió la prueba de cizallamiento debido a que reproduce mejor la dinámica mandibular ^14^. Diversos estudios similares también utilizaron esta prueba para medir la resistencia adhesiva entre diferentes materiales restauradores y sustratos dentarios ^15^.

La prueba de cizallamiento se define como la fuerza vectorial aplicada a un cuerpo en la que se trata de desplazar a otro cuerpo en sentido vertical ^16^. En este estudio, se utilizaron dientes bovinos ampliamente aceptados en los estudios *in vitro*, aunque con una resistencia adhesiva menor que en dientes humanos. Nakamishi *et al*. ^17^ y Yuk *et al*. ^18^ observaron que los valores promedios de resistencia adhesiva en dientes bovinos fueron ligeramente más bajos debido a que la tensión superficial crítica es más baja. 

La técnica adhesiva convencional en dentina se considera inestable ^19^, una de las razones es la acción de las MMP (metaloproteinasas) en la dentina producidas por los odontoblastos durante la secreción dentinaria. Después de la mineralización de la matriz de colágeno, las preformas inactivas de MMP quedan atrapadas en la matriz calcificada y pueden exponerse y activarse durante el proceso de caries en la dentina ^20^. Settembrini *et al*. recomiendan usar el desinfectante cavitario después del grabado ácido, porque elimina la capa de frotis de bacterias ^21^ que pueden invadir los túbulos dentinarios a una profundidad de 1100 mm, a diferencia de los desinfectantes cavitarios, que no penetran más de 130 mm en la dentina (^22^). En este estudio, se observó un aumento significativo de resistencia adhesiva con CHX en comparación con el control. Al igual que otros estudios previos ^23^^,^^24^, se observó que la unión en el grupo de CHX fue significativamente mayor al control después de 12 meses. Por otra parte, Say *et al*. ^25^ concluyeron que la CHX no afectó la fuerza de adhesión. 

Estudios como Gunaydin *et al*. ^26^ difieren de nuestros resultados, pues informan que la resistencia adhesiva de las muestras sin pretratamiento con CHX eran significativamente más altas que las pretratadas. Otro estudio, realizado por Di Hipólito *et al*. ^27^ evaluaron la resistencia adhesiva de cementos autoadhesivos a dentina pretratada con CHX, lo cual demuestra que la resistencia adhesiva microtensional en los grupos control fue significativamente mayor que en los grupos tratados con CHX.

Además, Vieira y Da Silva ^28^ evaluaron el efecto de la CHX en la resistencia adhesiva de RC a dentina en dientes primarios, y demostraron que la aplicación de CHX disminuye la resistencia adhesiva. Asimismo, Fernandez *et al*. ^29^ refirieron que la CHX no se eliminó por el lavado de la dentina, lo cual plantea la hipótesis de que los túbulos dentinarios quedarían ocupados por moléculas de CHX y residuos en la dentina. Esto podría interferir en la infiltración de los monómeros resinosos del adhesivo en la dentina.

En los resultados respecto del grupo de TFD, se observó un aumento en la resistencia adhesiva en comparación con el grupo control, como en el estudio de Celik *et al*. ^30^, en el que se usó la TFD y se encontró que la resistencia adhesiva aumenta de manera significativa en comparación con el grupo control. Pereira *et al*. ^31^ informaron que las muestras irradiadas con láser Er:YAG mostraron valores de fuerza de unión más altos que sus respectivos controles. Por otra parte, en el estudio de Yazici *et al*. ^32^ observaron que los valores de resistencia adhesiva no difirieron después de la aplicación de láser Nd:YAG en dentina, para el adhesivo de autograbado probado. Por el contrario, en un estudio realizado por Ferreira *et al*. ^33^ mostraron que la irradiación con láser Er:YAG y Er,Cr:YSGG debilitó la adhesión en dentina. Es más, el aumento del tiempo de grabado no modificó la fuerza de la unión del adhesivo a la dentina irradiada.

Los resultados entre la TFD y CHX no presentaron diferencia significativa al igual que el estudio de Dalkilic *et al*. ^34^ donde informaron que el láser Nd:YAG y la CHX al 2% se pueden utilizar como desinfectantes en la dentina antes del protocolo adhesivo.

Por el contrario, en el estudio de Oznurhan *et al*. ^35^ encontraron que los dientes pretratados con TFD antes del protocolo adhesivo mostraban una resistencia adhesiva más alta que el grupo CHX. Asimismo, Deeb *et al*. ^36^ informaron que la CHX presentó mayor resistencia adhesiva en comparación a la TFD. Por otra parte, en la revisión sistemática de Coelho *et al*. ^37^, que incluyó nueve estudios de láser, se encontró un mantenimiento o aumento en los valores de la fuerza de unión, dado que se utilizan diferentes láseres, con diferentes parámetros y en diferentes condiciones.

El resultado de nuestro estudio puede deberse a que la TFD no altera la superficie de la preparación dental en una cavidad, lo cual permite una adhesión efectiva; podríamos inferir que se forma una adecuada capa híbrida. El aporte de este estudio da a conocer al clínico que la resistencia adhesiva de la resina a dentina tratada previamente con TFD y CHX podrían ser seguras al mostrar valores altos de resistencia adhesiva. Se debe considerar que la TFD es poco común en el ámbito odontológico ^35^.

## CONCLUSIONES

La terapia con clorhexidina y con láser diodo 660 nm mostraron resistencias adhesivas estadísticamente similares, pero superiores frente al grupo control.

## Fuente de financiamiento:

Autofinanciado.
